# Healthy Operator 4.0: A Human Cyber–Physical System Architecture for Smart Workplaces

**DOI:** 10.3390/s20072011

**Published:** 2020-04-03

**Authors:** Shengjing Sun, Xiaochen Zheng, Bing Gong, Jorge García Paredes, Joaquín Ordieres-Meré

**Affiliations:** 1 Escuela Técnica Superior de Ingenieros Industriales (ETSII), Universidad Politécnica de Madrid, José Gutiérrez Abascal 2, 28006 Madrid, Spain; shengjing.sun@alumnos.upm.es (S.S.); xiaochen.zheng@epfl.ch (X.Z.); j.gparedes@alumnos.upm.es (J.G.P.); 2 Exposure, Epidemiology, and Risk Program, Department of Environmental Health, Harvard T.H. Chan School of Public Health, Boston, MA 02115, USA; 3 ICT for Sustainable Manufacturing, SCI-STI-DK, École polytechnique fédérale de Lausanne (EPFL), 1015 Lausanne, Switzerland; 4 Jülich Supercomputing Center, Forschungszentrum Jülich GmbH, Wilhelm-Wohnen-Str, 52428 Jülich, Germany; b.gong@fz-juelich.de

**Keywords:** healthy operator 4.0, human–cyber–physical system, industrial internet of things, industry 4.0, smart workplaces

## Abstract

Recent advances in technology have empowered the widespread application of cyber–physical systems in manufacturing and fostered the Industry 4.0 paradigm. In the factories of the future, it is possible that all items, including operators, will be equipped with integrated communication and data processing capabilities. Operators can become part of the smart manufacturing systems, and this fosters a paradigm shift from independent automated and human activities to human–cyber–physical systems (HCPSs). In this context, a Healthy Operator 4.0 (HO4.0) concept was proposed, based on a systemic view of the Industrial Internet of Things (IIoT) and wearable technology. For the implementation of this relatively new concept, we constructed a unified architecture to support the integration of different enabling technologies. We designed an implementation model to facilitate the practical application of this concept in industry. The main enabling technologies of the model are introduced afterward. In addition, a prototype system was developed, and relevant experiments were conducted to demonstrate the feasibility of the proposed system architecture and the implementation framework, as well as some of the derived benefits.

## 1. Introduction

The continuous technological innovations in the domains of information technology (IT), the Internet of Things (IoT), and artificial intelligence (AI), among others, have significantly changed production systems [1,2,3]. Recent advances of these technologies have enabled a systematical implementation of cyber–physical systems (CPS) in manufacturing, which has significantly improved the efficiency of production systems and made them perform more resiliently and collaboratively. These advanced technologies are transforming the manufacturing industry to the Industry 4.0 paradigm [4,5].

In the Industry 4.0 era, all items in a smart factory will be equipped with integrated communication and certain data processing capabilities, by the so called Industrial Internet of Things (IIoT). The IIoT scenario enables a close connection between the physical and digital worlds, and the paradigm of cyber–physical equivalence or digital twin emerges [6], where the vision of the digital twin itself refers to a comprehensive physical and functional description of a component, product, or system, which includes more or less all information that could be useful in all lifecycle phases [7]. This represents a seamless integration between both worlds, meaning that the digital part can virtually replicate the behavior of the physical counterpart and, henceforth, be used to create new added value services in both directions.

The smart “artifacts”, including people, will be connected to the CPS [8,9], which is different from computer integrated manufacturing (CIM), as the intention of Industry 4.0 paradigm is not creating unmanned production facilities. In contrast, it provides a great opportunity for operators to become part of the smart manufacturing systems in such a way where the individual skills and talents of the operators can be better realized [10,11,12].

The advanced technologies, such as IoT and CPS, in the Industry 4.0 paradigm provide new forms of interaction between operators and machines that will produce new intelligent workforces and will bring significant impacts on the nature of manufacturing [13,14]. Human-centricity is one of the most critical focuses in the transformation towards Industry 4.0. It allows for a paradigm shift from independent, automated, and human activities to human–cyber–physical systems (HCPSs) where machines are not designed to replace the skills and talents of humans, but rather to co-exist with, and to assist humans in being more efficient and effective [15,16]. A clear opportunity in such a path is to align the human central view in the decisions for process improvement, where improvement in the classical lean management, e.g., (CPD)${}_{n}$A [17], was performed through a strategy enabling taking advantage of the IoT devices, and looking to increase the knowledge analysis capabilities of the responsible worker. Therefore, the aims of HCPSs can be described [12,15] as:

- Empowering people to dynamically interact with machines in both the cyber and physical worlds to fit the operators’ cognitive and physical needs supported by intelligent human–computer interaction techniques;
- Improving the physical, sensing, and cognitive capabilities of people taking advantages from diverse technologies, such as IoT and wearable technologies.

On the basis of HCPSs, the concept of Operator 4.0 was proposed, aiming at improving the cooperation between humans and machines [13,15]. Operator 4.0 represents “a new design and engineering philosophy for adaptive production systems where the focus is on treating automation as a further enhancement of the human’s physical, sensorial, and cognitive capabilities” [15].

According to different enabling technologies and targeting aspects, Operator 4.0 can be empowered by different technologies [12,13], such as,

- Virtual operator, enabled by virtual reality (VR)/augmented reality (AR) [18],
- Super-strength operator, enabled by exoskeletons [19],
- Smarter operator, enabled by intelligent personal assistant (IPA)-based solutions [20],
- Healthy operator, enabled by wearable technologies combined with advanced data analytic techniques [21],
- etc.

As one of the core sub-types of Operator 4.0, the Healthy Operator 4.0 (HO4.0) aims to address the concerns regarding increasing workforce stress levels, the state of psycho-social health [22,23], and the new potential physical risks [24,25] in the cyber–physical production environments caused by the introduction of new Industry 4.0 technologies, including autonomous and collaborative robots, AR, and VR.

Although occupational health has been a popular topic for decades in both academic research and industrial applications, it has rarely had the advantage of real time quantitative measurements, mainly due to the limited capabilities for directly measuring such factors [21,26,27,28,29,30,31].

The recent advances in sensing technologies and IoT, especially wearable technologies, provide new solutions for real-time monitoring of an operator’s activities, locations, vital signs, etc., as well as the status of the surrounding workplace environment [30,31,32]. These technologies make it possible to develop health-related applications, such as alerting operators of possible exposure to hazardous environments, avoiding collisions with moving heavy equipment, and preventing anti-ergonomic body movements and postures [21,33]. However, in current industrial practice, most applications are developed in isolated circumstances aimed at addressing specific problems. Therefore, there is a gap in creating human-centered systems able to promote operators’ learning context not only relying on single parameters but also providing a meaningful articulated set of relevant parameters both in the short and long term.

To cope with this challenge, a unified architecture for HO4.0 is required to support the integration of different enabling technologies, thus guiding the implementation of this concept in the context of occupational safety and health. The focus of the paper is established to:

- Formally define the HO4.0 concept, by carefully analyzing the contributions from previous research in relevant fields,
- Propose a HO4.0 application framework enabling relevant data integration in highly dynamic work environments, supported by the IIoT and wearable technology. According to the definitions, we presented a multi-layer architecture and an implementation framework to guide the their application in industry;
- Provide a prototype system, developed according to the proposed framework, where the assessment of its capabilities can be derived; and
- Conduct experiments to verify the capabilities of the prototype system to provide new knowledge on real applications.

From the methodological point of view, this study followed the critical action research method, in which the researcher and a field operator collaborated in the diagnosis of the problem and in the development of a solution based on the diagnosis [34]. Critical action research is based on the analysis, action, evaluation, and critical analysis of practices based on collected data, in order to introduce improvements in the relevant practices. This type of research is facilitated by the participation and collaboration of a number of individuals with a common purpose where the research focuses on specific situations and their context.

The rest of this paper is organized as follows: Section 2 introduces the formal definition for HO4.0, enabling us to build over the proposed framework, in Section 3. Section 4 introduces the application case supporting the prototype creation. The experiments and outcomes are presented in Section 5. Then Section 6 summarizes the findings and provides our interpretation in the context of the proposed HO4.0 framework. Finally, Section 7 provides a more strategic perspective of the knowledge gains, including the limitations and further research directions.

## 2. HO4.0 Concept

As the sub-type of Operator 4.0, the HO4.0 concept was first envisioned in [12], where it was roughly depicted as *Operator + Wearable Tracker = Healthy Operator (physical and cognitive interaction)*. In this stage, individual wearable devices, such as smartwatches, were used to track an operator’s health-related metrics. However, an isolated and separate monitoring dimension can not fully represent comprehensive health aspects. Therefore, a unified health view of the operator is needed to enable holistic workforce health management and analytics. In this study, aligned with the Operator 4.0 definition [13,15], and extending the former concept, the HO4.0 is formally defined as a system focused on health and operator well-being, looking to facilitate the operators’ empowerment by enabling relevant knowledge creation, including modeling their behaviors.

It is relevant that our adopted perspective differs from the one chosen in [12,21], where the the concept was closer to the operator. It was introduced as a specific type or vision for the Operator 4.0 concept. Instead, the ambition here was to consider the relevant information gathered, when appropriately fused, as a digital image of the operator’s behavior, and due to this, the digital twin approach comes in naturally. To facilitate the integration of concepts, the natural approach is to consider the HO4.0 as a system instead of a type of operator. Under this interpretation, the HO4.0 can be also presented as a virtual system: the HO4.0 digital twin, gathering all the health related information from operators and potentially enabling the learning of rules from the different behaviors. This does include benchmarks regarding the health impact of different operator routines, etc.

The HO4.0 digital twin is powered by IIoT networks, wearable technologies, ambient intelligence, and modeling technologies. It not only enables real-time health risk information, e.g., risk alert, but also make it possible to simulate the future behavior of operators; to infer and forecast the evolution from their behavior at mid-long term ranges, aiming to reduce the operator’s cognitive and physical workload, and increase the operator’s well-being such as Occupational Safety and Health (OSH), job satisfaction, work-related affect, and enhance the workforce productivity in the Industry 4.0 context.

## 3. HO4.0 Framework

The proposed framework has two main components, one is the architecture, and the other involves the required technologies.

### 3.1. Proposed Architecture

**Figure 1:**
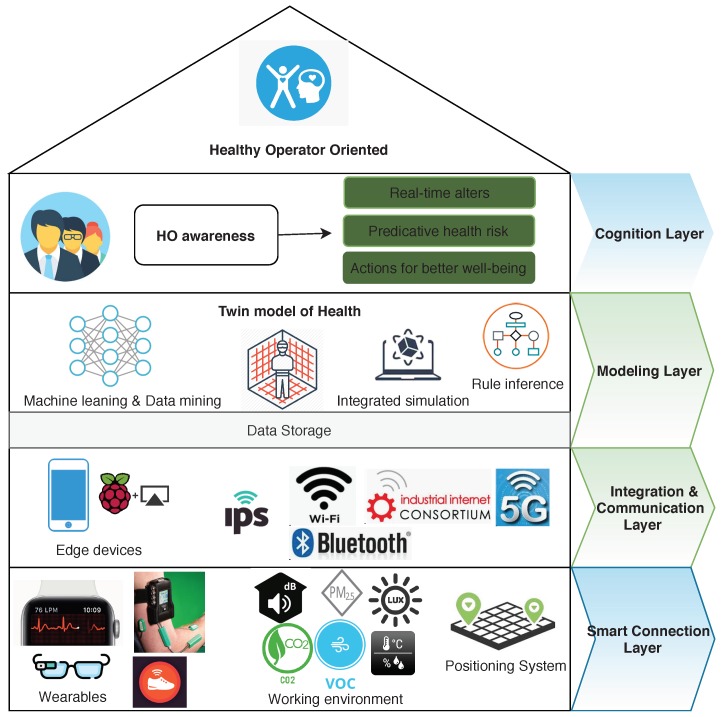
Healthy Operator 4.0 (HO4.0) architecture.

A traditional CPS structure usually includes five levels (5C structure), including Smart Connection, Data-to-information Conversion, Cyber, Cognition and Configuration [4]. Based on the Operator 4.0 definition, we adapted the 5C structure of CPS and designed a four-level architecture for HO4.0, compatible with the 5C CPS structure, as the proposed architecture shown in Figure 1 is composed of the Smart Connection layer, the Integration and Communication layer, the Modeling layer, and the Cognition layer.

- **Smart Connection layer:** Accurate and reliable data from operators, machines, the ambient environment, and other parts of the production system are the foundation of the HO4.0 system. The sensing layer collected these data utilizing wearable devices, ambient sensors, and the industrial sensors of manufacturing systems etc. Wearable devices, such as smart watches, smart bands, smart glasses, smart shoes, and smart helmets, contain a variety of sensors that can measure the operator’s location, movements, heart rate, blood pressure, body temperature, concentration level (e.g., fatigue), foot pressure, etc. Stationary or portable ambient sensors, such as air quality, thermal (temperature and humidity) sensors, and acoustic and light sensors, are used to monitor the conditions of the workplace environment surroundings, such as the indoor air quality level, temperature, humidity, pollutant concentration, light intensity, and noise levels. The indoor positioning system (IPS) was adopted to detect the movement and location of the operators, vehicles, work pieces, machines, etc. in a workplace.
- **Integration and Communication layer:** This layer was mainly composed of edge computing devices or gateways, which could include a smartphone, tablet, router, or single-board computer (SBC) etc. This layer was required due to the limitations of the size, power supply, and computing capabilities of most wearable devices and ambient sensors from the sensing layer. They are not capable of performing operations such as data filtering and integration. This layer aimed to filter and integrate these diverse data, and if necessary, to convert and pre-process the raw data based on different data modeling approaches. The sensors used in the sensing layer are from many domains, e.g., monitoring human psychological behaviors and detecting environmental conditions. This layer also facilitated the data uploading to the server storage for further data analysis. The edge computing device in this layer can be applied to provide real-time health warnings that are critical for a human that interacts with machines and HCPSs.
- **Modeling layer:** The health related data was fused and modeled in this layer. The twin model of operator health was created and relevant simulations can be run to apply the derived high lever rules. This is typically conducted in a local or remote/cloud server with a higher computing capability that is able to care about data streams from different communication devices. Semantic data description enables the interoperability and usage of semantic models, and knowledge graphs might also be employed to facilitate the knowledge extraction. Supported by advanced machine learning techniques and semantic-driven data fusion, useful knowledge, such as risk alerts, improved advice, and rules, can be extracted.
- **Cognition layer:** Based on the results from the model layer, the cognition layer can provide hints and insights from cyber space to physical space and acts as a monitoring system for the preventive decisions from operators, machines, or ambient environments. On this layer, analyzing the results can support the decision-making and can be presented with proper data visualization techniques. Certain user interfaces, applets, or web-based services can be also implemented. The alerts, advice, and orders can be delivered to different stakeholders to help them conduct corresponding actions. The target is to ensure work–life health, safety, and satisfaction.

### 3.2. Enabling Technologies

The successful implementation of HO4.0 architecture requires the support of a series of advanced enabling technologies. Some of the ones that were mentioned in the model and used in the prototype are introduced as follows.

- **Wearable technology:** Wearable technology or wearable computing is the study or practice of inventing, designing, building, or using miniature body-worn computational and sensory devices [35]. With the rapid development of sensing technologies, various types of sensors can be embedded in wearable devices. According to their features, they can be divided into four major groups [36]:
  1. Environmental sensors, such as light sensors, temperature sensors, sound sensors, humidity sensors, air quality sensors (e.g., CO_2_ sensors, particulate matter (PM) sensors, and volatile organic components (VOCs) sensors), and barometric sensors.
  2. Biosensors, including body temperature sensors, heart-rate-monitoring sensors, electrocardiogram (ECG), electroencephalography (EEG), electromyography (EMG) sensors, blood pressure sensors, galvanic skin response (GSR) sensors, eye tracking, weight insole, and glucose level sensors.
  3. Location tracking sensors, such as GPS, altimeters, magnetometers, compasses, and accelerometers.
  4. Other sensors, including camera sensors, communication sensors, ultrasonic sensors, infrared receiver (IR), sensors.
  The application of wearable technologies in HCPSs provides rich information related to the operators and the surrounding environment.
- **Indoor Positioning System:** IPSs locate and track objects within a closed environment usually based on triangulation and multilateration methods using light, ultrasound, or radio signals to provide positional information [37]. The tracking of operators and production equipment can be significantly improved by the application of IPS [13]. Several wireless technologies might be used in IPS depending on the application situations by means of different positioning algorithms [38], such as Global Positioning System (GPS), Radio Frequency IDentification (RFID), Cellular networks, Ultra-wideband (UWB), wireless local area network (WLAN), Bluetooth etc. The adoption of IPS makes possible of a context-aware system for HO4.0 to help operators monitor the locations of machines, vehicles and workpieces to improve productivity and avoid potential collision risks.
- **Ambient environment monitoring:** The condition of the ambient environment plays a crucial role in a HO4.0 system. It may impact the operators’ working performance or even harm their health. For example, indoor air pollution is one of the leading environmental risks, and indoor air quality (IAQ) is proven to have significant impacts on human comfort, health, and performance [29]. The advancement of low-cost IoT sensors, in recent years, has enabled the use of wireless communications and computing for interacting with the physical world. These sensors can measure indoor environmental parameters including IAQ, comfort, lighting, and acoustic conditions [39,40,41]. Many of these low-cost ambient sensors allow portable deployment and different sensors can be easily packed in one board to fit the requirements of different application scenarios. The data generated by such sensors can be collected by certain edge computing devices, such as single board computers (SBC). As an example, an ambient environment monitoring system based on a sensor island and a Raspberry Pi SBC was developed in a previous study [29]. The sensor island used in this system was composed of nine different ambient sensors to monitor different aspects of the ambient environment. More details of this system are explained in the prototyping section.
- **Knowledge engineering:** A semantic data model is a high-level representation of knowledge. It is designed to capture the meaning of data attributes and relationships, and obtain the meaning of each instance in an application context. The standard data model and knowledge engineering facilitates information exchange and data interpretation in a very heterogeneous data sources. Fusion and integration data based on rich semantic context gravitates toward applications, such as effective decision making processes, by fully making use of the various heterogeneous data. Ontologies, stemming from the Semantic Web, are used to define standard and common terms, vocabulary, and relationships for a particular area/domain. Aligning on common interpretations through ontologies, data/knowledge graphs can be generated to refer to the relationships among different data sources from different domains. Existing or adapted ontologies [42,43] are recommended to be used for semantic modeling aiming to derive action rules toward the operator’s health management.
- **Machine learning (ML):** The data gathered from the sensing layer can contain valuable information, which is the core of the HO4.0 system. Indeed, when scaled up appropriately, it can become a large volume of data; whereas, a global analysis can help in providing better interpretations for the impact of different behaviors. Therefore, advanced data modeling tools, such as machine learning, can be useful to create or emphasize such new knowledge. The wide applications of the IoT have promoted the concept of big data, which on the other hand, has fostered the explosive involvement of the advanced machine learning techniques represented by deep learning. Despite the great achievement of machine learning in many fields, its power has not been fully and systematically explored in the industry sector, particularly in the human-centric systems. Here the benefits from the adopted concept for HO4.0, such as the system proposed in this research (which enables the digital twin approach) facilitates modeling capabilities to detect patterns and to add value in the real world for human-centric applications. For example, deep learning can be used to monitor an operator’s activities based on the motion data collected from wearable devices [9,44,45], but when combined with health related information and environmental conditions, this can provide a health index for such activities. In addition to the self learning process based on the real time data, the inter-comparison between workers would help to better understand the physiological and mental demands, but it can also help to find different patterns and to learn about the sustainability of them for the long term [46].

## 4. The Application Case and Experiments

To validate the proposed architecture, an application case was carried out, enabling the construction of a prototype helping with different practical implementations.

### 4.1. Case Selection

The case is connected with a logistic intra-facility, where the interest was in obtaining health related knowledge of crane operators in their working environment, who were in charge of loading trucks that moved manufactured steel rebar to build singular concrete structures.

**Figure 2:**
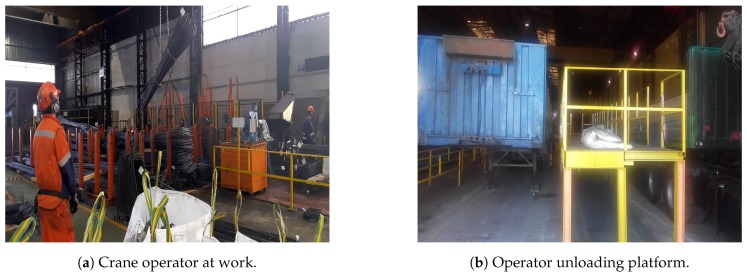
Crane operator working conditions.

Figure 2a shows how an operator moved around, looking for the pieces to be loaded, then attaching them and loading them into the trucks, where they went up and down a specific platform as presented in Figure 2b, which was designated to facilitate access to the truck itself and to maintain awareness on how the material was placed inside the truck. The preparation of the materials on trucks must be carefully planned as the unload process typically needs to visit different construction sectors to deliver specific sets of rebar. Therefore, a specific disposal sequence of items as per the layers in the truck must also be carefully planned and is prone to errors and stressful situations, where there are doubts about what was actually loaded into the truck. This is even more sensitive for sets involving small numbers of light rebar, where weight variations are not informative enough about errors in the loading process.

Outside of their specific job, the crane operator was working in a rather complex environment, with potential environmental quality concerns. This environment was a plant that manufactures steel rebar, where the production of specific items by cutting, blending, and welding may affect the air quality. Due to the working environment, as shown in Figure 2a, acoustic and illumination aspects were be other health threats.

Due to the different working conditions for crane operators and their complex mobility schema, their mental working environment, and physical workload, and because of the continuous over-pressure to quickly deliver products to trucks, we determined that these workers were suitable for inclusion in the validation for the HO 4.0 architecture.

### 4.2. Sensing Layer

In this application, based on the specific characteristics of the workers, the sensing layer was designed to include different dimensions, including the:

- **Environmental conditions**, including temperature, humidity, and noise, as they can impact the operators’ health, as well as their performance.
- **Heart rate**, as related to their physical or mental effort.
- **Blood Pressure**, also as related to their physical or mental effort.
- **Arm angle**, referring to the horizontal line, as a reference for ergonomic working activities.
- **Position**, as a reference for understanding the movements and knowledge requirements in order to complete the work.
- **Steps**, as a reference for the physical effort.
- **Crane position**, in relation to the crane operator movements, allowing us to identify the effective working time.

Therefore, different sensing devices need to be attached to the crane operator to collect the information in relevant frequency patterns, although they must be as lightweight as possible, in order to be feasible for daily operations.

**Figure 3:**
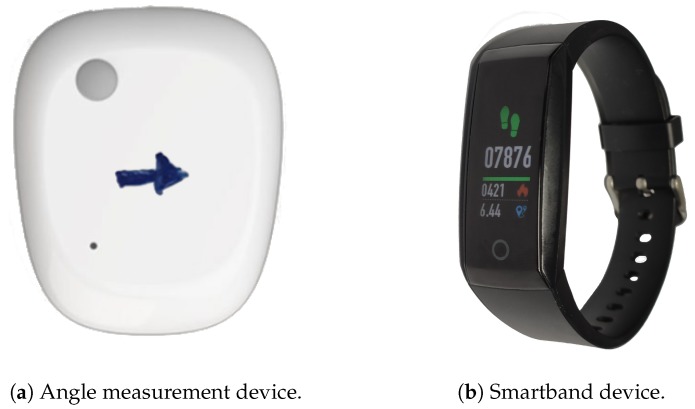
Wearable devices used in this application.

For the prototype, we decided to include three devices attached to the crane operators and one environmental station deployed on the shopfloor. Two of them were based on low cost devices, a MetaSensor (see Figure 3a) from mbientlab (https://mbientlab.com/store/metamotionr/), enabled us to monitor angles and acceleration and was used to track the arm angle through time. Another sensor we included was a smartband from HBand (see Figure 3b), able to track the heart rate, blood pressure, and steps.

For positioning the crane operator and the crane hook, we decided to use Ultra-wideband (UWB) technology with six stations able to track those positions [47]. The technology named $uRTLS^{TM}$, is a high precision real-time location solution (RTLS) based on UWB technology. The solution used several channels available at 3–7 GHz using the Decawave UWB chipset in compliance with IEEE 802.15.4.

The indoor environmental quality substantially affects a operator’s health, comfort, working performance, and well-being. In this study, the working environment for operators was monitored with an IoT based environmental quality monitoring system developed by [29]. The measured indoor environment conditions included: the chemical environmental parameters: particulate matter (PM), formaldehyde (HCHO), Total Volatile Organic Compounds (TVOC), benzene (C_6_H_6_), carbon dioxide (CO_2_), carbon monoxide (CO), ozone (O_3_), nitrogen dioxide (NO_2_), and the physical parameters: temperature, humidity, illumination, and noise.

Although these specific devices were selected for this implementation, without a lack of generalization, certain other devices, such as smart insoles, smart helmets, and skin conductivity sensors, could be also adequate, depending on the application case under consideration.

### 4.3. Integration and Communication Layer

In this application, the usage of several technologies were required in order to properly handle the data streams. There were data collected from the factory that benefited from using the existing extranet to upload the datasets to the data repository. This is the case for the environmental monitoring station, which was based on a raspberry pi delivering the data throughout its internal network card. This was also the case for the positioning system, which was acquired from the tracktio company (https://tracktio.com/). This system had its own data repository, therefore an additional middleware was required in order to enable data integration.

**Figure 4:**
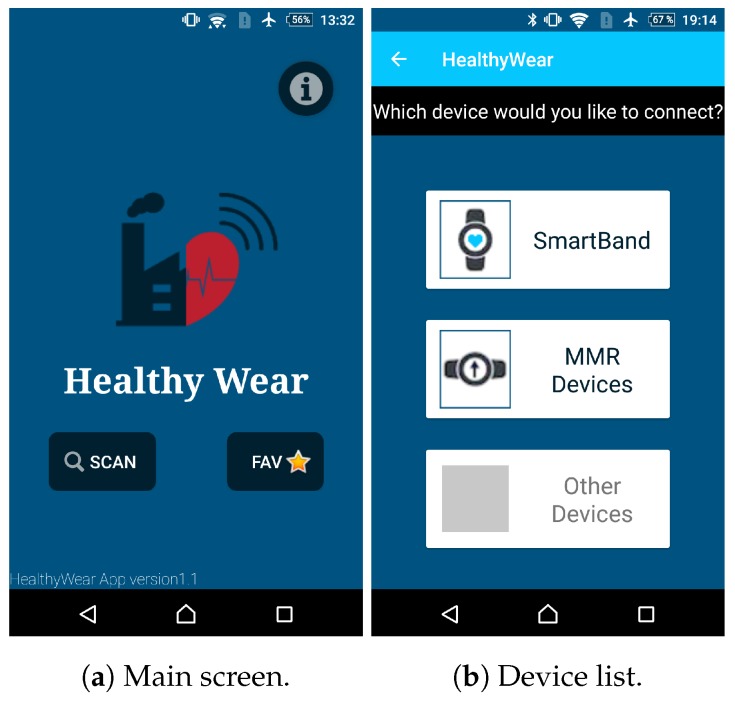
The app developed for data collection.

There were other data streams that were collected only by bluetooth, as the devices only had this interface. To handle these streams, an Android based app was developed [48], making it convenient to integrate the features and enabling a flexible data collection architecture [49]. Figure 4 presents the main details for this application, which collected data according to a specific time sequence. For this application, a minute-based frequency was adopted for health and environmental parameters, and a second-based frequency for the position, which provided convenient granularity for the learning application. Indeed, it was relevant that the developed app showed real time values and informed the operator regarding significant values. Although it was possible to order smartband vibrations when specific conditions were met, this was not activated as it could distract the operators from their main task and possibly become an unacceptable safety risk.

**Figure 5:**
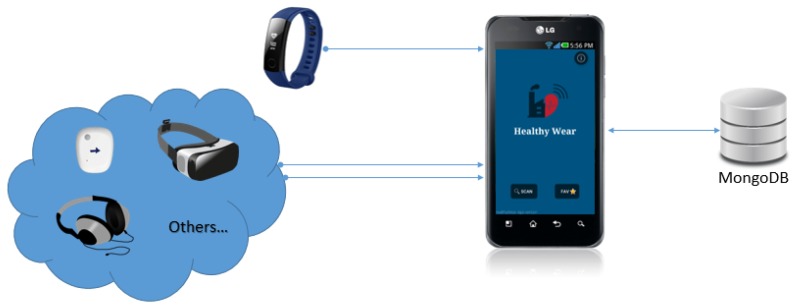
Overview of the applied wearable data flow.

The overall context for the data handling of the applied wearable in this study is presented in Figure 5.

### 4.4. Modeling Layer

With all the data streams ingested, this layer facilitated the fusion and time alignment as per the device media access control (MAC) address and time. Therefore, the record-sets were derived providing context for the digital twin, according to the chosen application case. The data fusion could integrate data streams that in some cases belonged to the same individual or in other cases belonged to properties of the area, affecting several operators on the shop-floor. The collected data streams allowed us to consider semantic annotation to enable a machine based data handling strategy.

The existing Vcard ontology for people [50] was used to model each operator. The indoor environmental quality ontology: AIR_POLLUTION_Onto, proposed by [51], was used to model the operator working environmental exposure, risk, and control applications. The physiology factor of the operator was semantically represented with HUMAN STRESS ONTOLOGY [52], considering the data sources steaming from the physiological parameters, such as the heart rate and blood pressure. The indoor navigation ontology introduced in the ILONA system [53] was applied for the indoor model positioning of the operators. Therefore, the considered set of references, based on the integration of such ontologies was as shown in Table 1.

Based on the specific interest, the annotated data streams were combined to create the contextual datasets, where their structure was not fixed at all. Instead, it was problem related. Therefore, when the interest was to analyze the similarity of the operating conditions and operator behavior through time, the application for building projectors with general non-linear dimension reduction was needed. Then, this layer will include different algorithms and tools, such as Principal component analysis (PCA), T-distributed Stochastic Neighbor Embedding (t-SNE), or Uniform Manifold Approximation and Projection (UMAP) dimension reduction techniques, which optimize a low-dimensional graph from twelve dimensions into two, in order to keep close records that are close in a higher dimensional space, and that preserved more of the global structure of the whole dataset.

When the interest is to look at specific behaviors that can be described by particular variations in variables, such variables were created and the ranges calculated. Based on those derived new variables, different Machine Learning techniques, such as association rules, can bring interesting rules explaining the causes for such behaviors.

When the case is to understand the behavior of operators through time, different regression algorithms were applied in order to forecast future trends, either by using the time series approach or the multidimensional one. Indeed, if the interest was to identify similar behavior between operators, different clustering algorithms were helpful, based on the medium term records of their digital twins.

The previous examples illustrated the strong possibilities that this layer can provide when the interest is to derive new knowledge for operators at different level of aggregation and for different time scales.

## 5. Results

**Figure 6:**
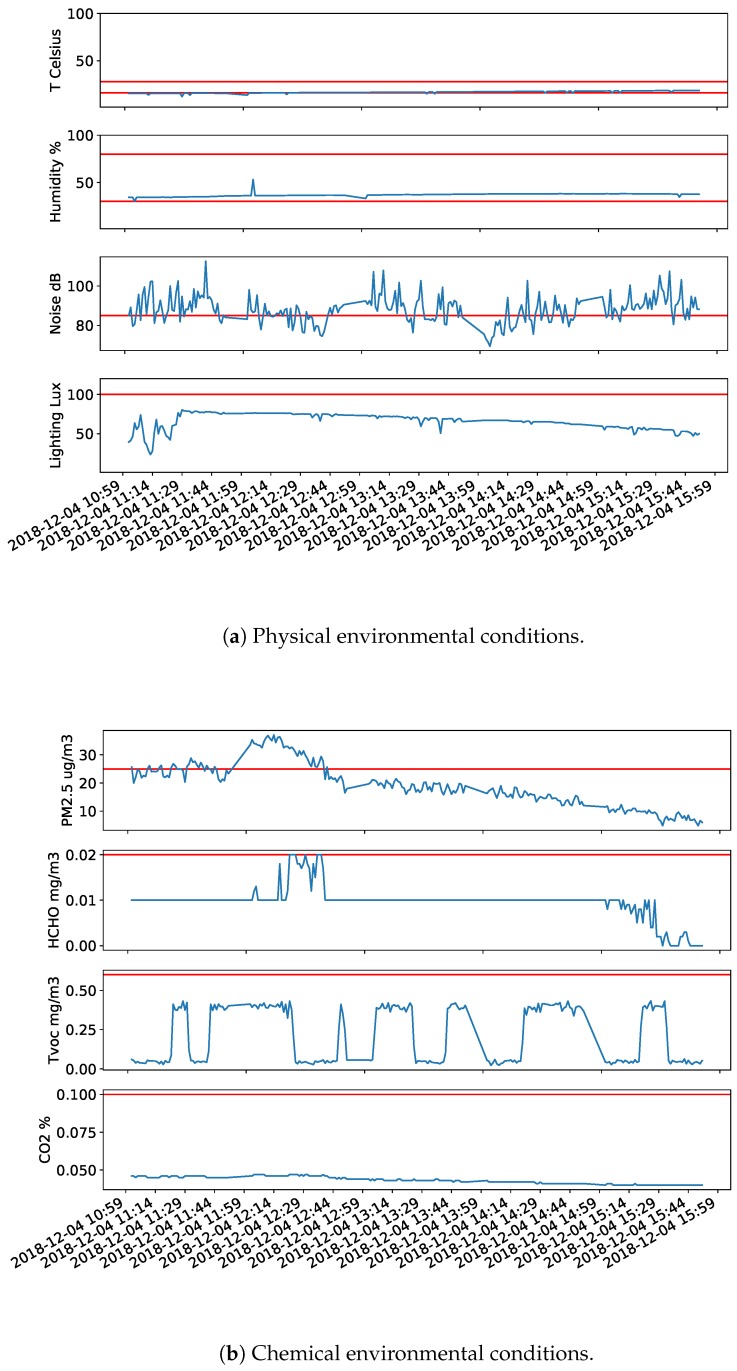
Operator exposure to the environmental condition on site. The red line refers to the threshold limit value (TLV).

The working environment of operators for this particular case study is dissatisfying as shown in Figure 6, due to different aspects where the actual exposition levels exceeded the threshold limit value (TLV).

The TLV of a environmental substance (chemical or physical) is defined to be a level to which a worker can be exposed day after day for a working lifetime without adverse effects. The recommended comfort TLVs for temperature and humidity are 16–28 °C and 30–80%, respectively, according to World Health Organization (WHO) [54]. For work that requires the perception of details, such as offices, sheet metal work, or bookbinding, the minimal luminance TLV is 100 Lux, which is defined by the European Union (EU) standard. The noise levels defined by WHO are 85–90 dB(A) daily average [55], the smallest value (85 dB (A)) was taken as the TLV to ensure acoustic health to the maximal extent. The TLV for PM2.5 and HCHO are a 25 µg/m${}^{3}$ average per day and a 0.016 ppm (0.02 mg/m${}^{3}$) average per 8 hours as defined by WHO [56] and National Institute for Occupational Safety and Health (NIOSH) [57], respectively. The TLV of CO_2_ (0.1%) and TVOC (0.60 mg/m${}^{3}$ average per 8 h) are based on a daily average, which obtained references from Circulate App: EnvCon [58]. The Circulate company set TLVs by taking references from China’s air-quality standards.

In this application case, the noise level was rather high consistently over time, as demonstrated in Figure 6a. In the vast majority of the working time, the noise level was higher than 85 dB(A) as defined by WHO [55], as the primary working activity is steel operations, which produces a huge amount of noise. Although operators are wear industrial headsets to protect their ears, constantly working in very noisy environments presents potential damage and hearing related problems, e.g., tinnitus. It also affects concentration at work, which was the case for crane operators. The environment was also rather dry and dim as depicted in Figure 6a (Humidity and Lighting). The chemical environmental conditions were inadequate as shown in Figure 6b. The PM2.5 and HCHO exceeded the contaminant level from time to time, and the TVOC is approached limits periodically. Therefore, these systems also help to identify dimensions where measures can be taken to improve the working environment, which would have a significant impact on the operators.

**Figure 7:**
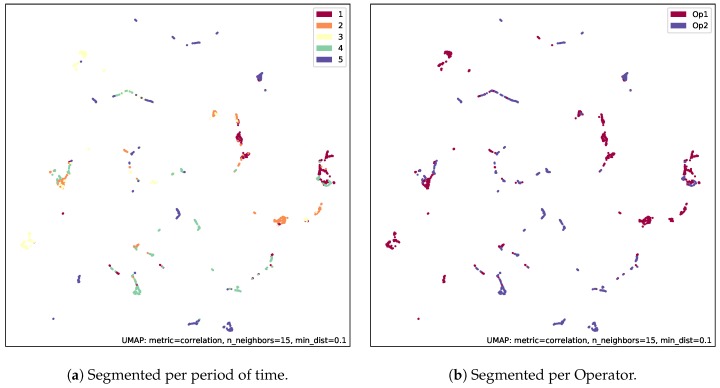
Projected patterns from the digital twin.

It is relevant to identify different clusters of behavior from the collected data as shown in Figure 7. In fact, this Figure presents a non-linear two dimensional projection from the originally 12-dimensional space of fused data from two different crane operators was embedded. It was decided to use the UMAP projection technique to provide a clear vision regarding the sample distribution where the inter-distance between samples was maintained and good preservation of the data’s global structure was granted [59].

The projection result, shown in Figure 7, depicts the whole dataset’s distributions and clusters phenomenon. Then, we decided to segment the whole dataset into five groups of the same length, to explore time dependency of clusters (see Figure 7a). The five groups were segmented via homogeneous distribution through time, and marked with different colors (labels 1–5). As far as the same color appears in different clusters, that means that they are not related to time mainly, but related to operator’s behaviour. In Figure 7b, the behaviors from the two monitored crane operators are presented. The projection of the whole dataset was also segmented into two groups: operator 1 and operator 2 marked with two different colors. It becomes clear that there is not operator specificity as per cluster, therefore the existence of many of them is due to intrinsic reasons. On the other side, different operators behaved in different ways, as different colors appeared in different clusters, although similarities due to the common activities were also there. Indeed, there was room to identify each of the clusters and to enrich the collection with larger datasets and, thus, derive knowledge from such clusters. The evolution of operator health such as stress level can be identified via clusters and amount of time on each, which makes it possible to adapt individual healthy requirement from management point of view. The objective measures can be taken, e.g., to allocate specific operator to less demanding trucks in order to provide better wellness or convenient recovery process.

The interest in the prototype was also to evaluate its functionality to derive specific knowledge when considering digital twins. In particular, the interest was to analyze the stress levels and the impacts. We decided to focus on situations where large differences between high and low blood pressure happen. This is because no matter whether an operator can suffer hypertension, an unwanted situation related to high levels of stress will have locally larger differences between the high and the low pressures, and the risk for cardiovascular disease, diabetes, chronic kidney disease, high cholesterol levels, and aneurysms will be larger [60]. A segmentation in different discrete categories between Low to Intense for the original variables as well for the derived ones (mainly differences and ranges), was carried out, including ΔBloodPressure, where the threshold for a large difference was adopted to be 45 mmHg. After the data preparation an a priori algorithm for the association rules discovery was applied, which provided some rules like those presented in Table 2.

Only relevant rules were highlighted as the algorithm discovered many others related to normal conditions, including normal environmental conditions and normal body indicators, which imply a normal range for ΔBloodPressure, etc. All of them had strong support and moderately high confidence however the lift values were close to 1. Indeed, the biggest interest was to check the rules with high confidence values, high lift figures, and moderate or low support. Those rules became excellent candidates to create knowledge (maximum confidence); however, due to their relatively low support, they remain mostly unknown.

During the validation of this prototype there was an unexpected high interest from the operators to obtain the knowledge about their parameters (to learn from their digital twins), and as far as it was possible and it was understood it could also provide benefits to the operators as they can become more aware of the reasons for their parameters; therefore, a significant effort was conducted to address the increase of awareness.

**Figure 8:**
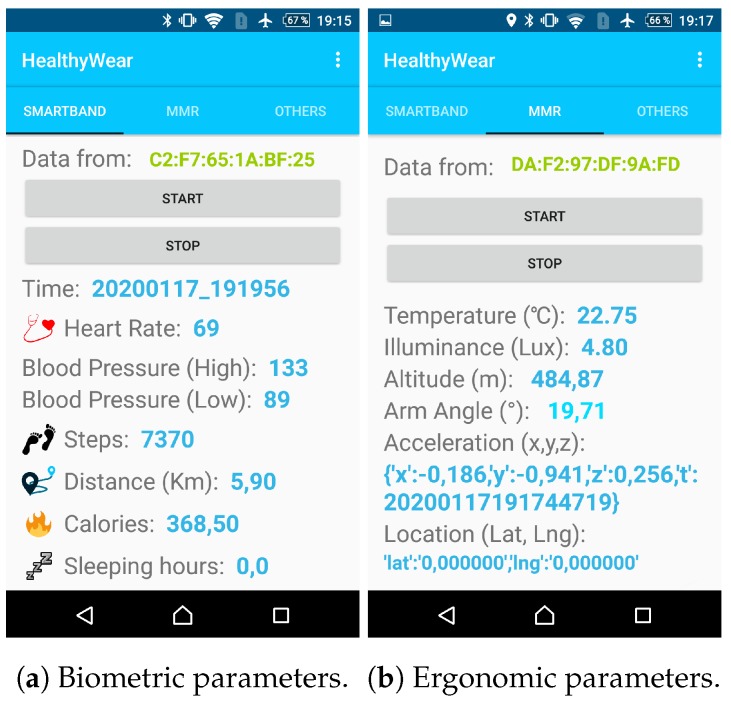
Informative phone screens.

The operator were able to have the real-time information of their health parameters, as shown in Figure 8.

**Figure 9:**
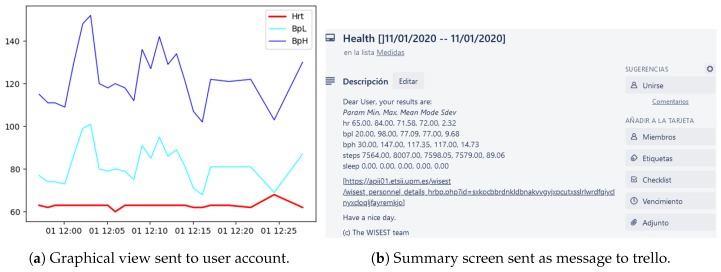
Informative user phone screens where time granularity can be configured.

**Figure 10:**
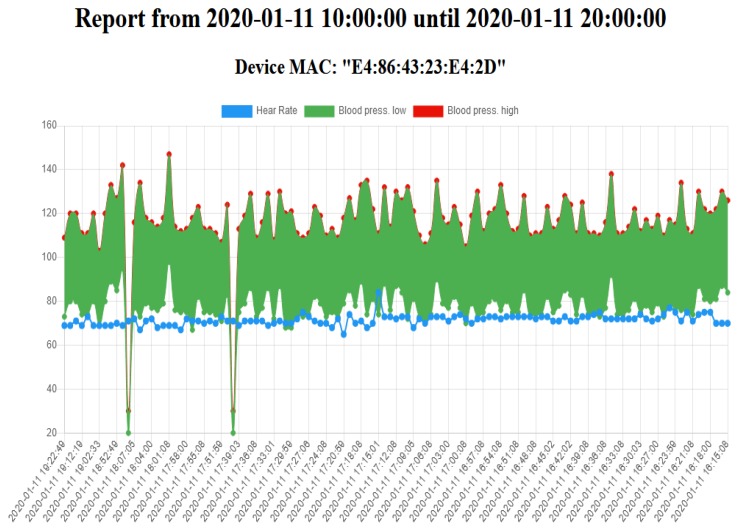
The graphical detailed view of the collected data at an individual level.

When lean management techniques are being used at the organization level, it can also help with the management of processes to communicate to the operators their figures on a daily basis, as described above for the (CPD)${}_{n}$A method [17]. Therefore, an application for the trello™system was created at the Cognition layer, where the detailed messages can be seen, as in Figure 9, bringing a way to deeply dig into the specific graphics Figure 10, making it possible to promote the operator self-learning process.

## 6. Discussion

The implementation of a prototype to validate the HO4.0 framework was performed successfully, where the layer structure helped in providing guidance to the required discussion in order to build the system. Data interconnection and interoperation further facilitated the making of health action decisions, aiming to improve the operator working conditions, and the strategies and actions were built on semantic-based data driver strategies.

The data from the sensing layer was collected via the data networking layer and was multimodal and heterogeneous when considering the different sensing instruments and data collection solutions. To make decisions, especially for the long-term policy in terms of health management, the data coming from different domains have to be interoperable and linked. We also needed to emphasize the meaning of each type of data streams, as well as the frequency of sampling, aligned with the relevant business model.

To further support decision making for health operator applications, different ontologies were applied in this study to make the data interoperable and semantically accessible while crossing domains. Although the already presented combination was retained for this prototype, there was no limitation other than to properly describe the entities and their inter-relationships to maintain a standard domain vocabulary and common understanding. Therefore, they can also be created on purpose. The metadata languages aligning with the ontology vocabulary convey that kind of shared information in a landscape of information management.

With the data inter-operation features and semantic annotation, a holistic view can be built up to enable the machine learning application to create new knowledge, as already presented. This can be operationalized and communicated looking to improve the operator health and safety.

Additionally, it is possible to combine the organizational analysis based on patterns with the additional value created from the operator level, by allowing them to be aware of their own values. This is another useful dimension of the HO4.0 concept, as awareness is a key element for empowerment [61].

## 7. Conclusions

Empowering people has significant implications for factories under the Industry 4.0 paradigm. Health, as one of main concerns affects operator’s job satisfaction and well-being. However, it was rarely investigated in the context of HCPSs. Aiming to reduce this gap, holistic view of operator health dimension was proposed, where the main contributions of this paper are summarized as follows:

- We explored the HO4.0 concept.
- Based on the classical five-level CPS structure, we proposed a four-layer framework for the implementation of the HO4.0 concept.
- We investigated of the enabling technologies for HO4.0.
- We developed a prototype system to showcase the application process of the proposed framework.
- We applied advanced machine learning and preparation with semantic engineering technologies to manage and analyze heterogeneous data from different sources, bringing this support to the digital twin perspective.

The research scope is not limited to the outcome of conducted experiments, but to provide an integrated framework providing vertical and horizontal integration capabilities. The proposed architecture supports easy integration of different data sources as from Industrial Internet of Things, wearables such as smart helmet, smart insoles, smart glasses, to name a few. From a practical perspective, it also successfully addresses the need for data integration from different devices, as manufacturers aim to provide their own data lakes, which are not flexible enough to allow user defined sampling frequency or convenient restful based data access. In addition, enhancing the capability for monitoring different devices with the same middleware (phone, tablet, etc.) including reconnecting capabilities, makes the data collection process stronger, which is really valuable in industrial environments.

The gathered data sources with the defined data fusion techniques, including semantic interoperability, produce multidimensional datasets, which are suitable to be analyzed in real time with advanced modeling technologies (big analytics). By learning from operators’ daily activities and parameters, inference about their evolution in long term perspective can be derived through their digital-twins. Such approach can have managerial value as adopted strategies can relay on transparent measurement system and they can better preserve knowledge, experience, and well-being for longer, as well as company enlarges its social sustainability dimension.

In addition, it was also possible to realize the high degree of interest that such applications open to workers themselves, and how it becomes a way of discussion between them, as they try to identify patterns and explanations. We have found it very interesting for using it in a smarter way of management, taking advantage of such interest in order to increase the empowerment and engagement of workers into their processes.

Further than that, based on the adopted methodology, we concluded that the designed framework for HO4.0, as depicted above, based on the HO4.0 concept, was adequate and easy to follow in the integration of different sources of data at any time.

As a limitation, the prototype was applied in only one company with a wide range of operator profiles, but where the size and culture can also have an impact, and these dimensions were not analyzed. Another limitation is based on appropriate sensor availability, as there are safety regulations that can limit the application of specific sensors, and there are a lack of sensors for certain tasks, such as human concentration. However, the range of applications was very large as the integration between the operator’s health and smart monitoring is just starting.

As a further research ambition, the prototype will be extended by integrating weight measurement through smart insoles, giving the information of the loads being carried by operators along the working time. This will be implemented by extending the ’Other devices’ option from the Android app, following the created implementation rules. Indeed, further research is needed to better categorize the identified clusters, its distribution and stability against sample size. Another research line is connected to the information that could be derived from the digital-twins when more data populate them. It seems to be very promising. Finally, but yet importantly, to connect the HO4.0 with production processes can bring a truly integrated management perspective which demands further investigations.

## Figures and Tables

**Table 1:** Data streams with semantic annotation.

Entity	Message
crane operator	{ “Ontology”:”https://bit.ly/2OxeEkO”,”object”:”vcard”,”fn”:”José Luis Fernández”, ”nickname”:”Jose”,”hasEmail”:”mailto:perpalper@gmail.com”, “Gender”:”Male”,”bday”:”1988-06-23”,”adr”:”José Gutiérrez Abascal 2, 28006, Madrid” }
environmental situation	{“Ontology”: “AIR_POLLUTION_Onto”, “object”: “airPollutants”, “deviceId”: “Airmonitor1”, “PM”: “25 ug$/m^{3}$”, “CO2”: “0.04%”, “VOC”: “0.4 mg$/m^{3}$, “NOX”: “0”, “Timestramp”: “09/08/2019 09:10:00”}
stress factor	{“Ontology”: “HUMAN STRESS ONTOLOGY”, “object”: “Measurements”, “deviceId”: “Hband1”, “Timestramp”: “09/08/2019 09:10:00”, “stressPhysiology”: [ {“heartRate”: “80”, “bloodPressureHigh”: “120”, “bloodPressureLow: “70”}]}
position	{“Ontology”: “ILONA”, “object”: “Position”, “deviceId”: “Crane1”, “Coordinate”: [{“x”:”40.342712”, “y”:”-3.123472”, “z”: “605.85”}], “Timestamp”: “09/12/2019 10:10:00”}

**Table 2:** The derived rules from the application of machine learning techniques looking at high differences in blood pressure and explaining the intense values.

Rule Antecedent	Confidence	Support	Lift
’Arm angle:UP FRONT’ AND ’High Blood Pressure:Intense’ AND ’Heart Rate:Moderate’	1.0	5.9%	3.86
’Sound Level:Noisy’ AND steps:Low AND Arm angle:UP FRONT AND ’High Blood Pressure:Intense’	1.0	6.27%	3.86
’steps:Low’ AND ’HCHO:Relative Moderate’ AND ’High Blood Pressure:Intense’	0.84	10.0%	3.85

## Abbreviations

The following abbreviations are used in this manuscript:

AI	Artificial Intelligence.
CIM	Computer Integrated Manufacturing.
CPS	Cyber–Physical Systems.
ECG	Electrocardiogram.
EEG	Electroencephalography.
EMG	Electromyography.
GPS	Positioning System.
HCPSs	Human-Cyber–Physical Systems.
HO4.0	Healthy Operator 4.0.
IAQ	Indoor Air Quality.
IIoT	Industrial Internet of Things.
IoT	Internet of Things.
IPA	Intelligent Personal Assistant.
IPS	Indoor Positioning System.
IR	Infrared Receiver.
IT	Information Technology.
MAC	Media Access Control
ML	Machine Learning
OSH	Occupational Safety and Health
RFID	Radio Frequency IDentification.
SBC	Single-Board Computer.
TLV	Threshold Limit Value
UWB	Ultra-wideband.
VR	Virtual Reality.
WLAN	wireless local area network.
